# Iatrogenic severe hyperglycemia due to parenteral administration of glucose in children – a case series

**DOI:** 10.1186/s13052-020-00939-9

**Published:** 2020-12-01

**Authors:** Nora Bruns, Anja Große Lordemann, Tobias Rasche, Jochen Meyburg, Marcus Krüger, Christian Wieg, Alexander Gratopp, Marc Hoppenz, Friedhelm Heitmann, Thomas Hoppen, Günther Löffler, Ursula Felderhoff-Müser, Christian Dohna-Schwake

**Affiliations:** 1 Department of Pediatrics I, Neonatology, Pediatric Intensive Care, and Pediatric Neurology, University Hospital Essen, Hufelandstr. 55, 45147 Essen, Germany; 2 Emergency Department, Children’s Hospital Hamburg-Altona, Bleickenallee 38, 22763 Hamburg, Germany; 3 Center for Childhood and Adolescent Medicine, University Hospital Heidelberg, Im Neuenheimer Feld 672, 69120 Heidelberg, Germany; 4 Department of Neonatology, Munich Clinic Campus Harlaching and Schwabing, Sanatoriumsplatz 2, 81545 Munich, Germany; 5 Department of Neonatology and Pediatric Intensive Care, Klinikum Aschaffenburg, Am Hasenkopf, 63739 Aschaffenburg, Germany; 6 Division of Pediatric Emergency and Intensive Care Medicine, Charité University Medical Center, Augustenburger Platz 1, 13353 Berlin, Germany; 7 Department of Neonatology and Pediatric Intensive Care Medicine, Children’s Hospital, Amsterdamer Str. 59, 50735 Cologne, Germany; 8 Department of Pediatrics, Klinikum Dortmund, Beurhausstr. 40, 44137 Dortmund, Germany; 9 Department of Pediatrics, Gemeinschaftsklinikum Mittelrhein, Koblenzer Str. 115-155, 56073 Koblenz, Germany; 10 Department of Pediatric and Neonatal Intensive Care, University Hospital of Saarland, Kirrberger Str. 100, 66421 Homburg, Germany

**Keywords:** Hyperglycemia, Iatrogenic, Pediatric, Treatment error, Parenteral feeding, Parenteral nutrition, Glucose, Administration

## Abstract

**Background:**

Iatrogenic severe hyperglycemia (ISH) caused by glucose-containing i.v. solution is a potentially fatal treatment error. The objective of this study was to investigate the causes, circumstances, course of disease, and complications of ISH > 300 mg/dl (16.7 mmol/l) in neonates and children.

**Methods:**

We emailed a survey to 105 neonatal and pediatric intensive care units in Germany, Austria, and Switzerland, asking to retrospectively report cases of ISH.

**Results:**

We received 11 reports about premature infants to children. Four patients (36%) had poor outcome: 2 died and 2 suffered persistent sequelae. The highest observed blood glucose was at median 983 mg/dl (54.6 mmol/l) (range 594–2240 mg/dl; 33.0–124.3 mmol/l) and median time to normoglycemia was 7 h (range 2–23). Blood glucose was higher and time to normoglycemia longer in patients with poor outcome. Invasive therapy was required in 73% (mechanical ventilation) and 50% (vasopressor therapy) of patients, respectively. Administration of insulin did not differ between outcome groups. Patients with poor outcome showed coma (100% vs. 40%) and seizures (75% vs. 29%) more frequently than those with good outcome.

**Conclusions:**

ISH is a severe condition with high morbidity and mortality. Further research to amplify the understanding of this condition is needed, but focus should largely be held on its prevention.

## Background

Hyperglycemia of different origins can result in complications including renal failure and neurological complications including seizures, coma, and brain edema [1–5]. A severe and potentially fatal condition is iatrogenic severe hyperglycemia (ISH) following administration of glucose-containing i.v. solution. It occurs due to human errors regarding prescription, preparation and administration of glucose-containing medication or parenteral nutrition.

Children might be especially susceptible to ISH, as they frequently receive glucose-containing i.v. solutions to meet their age-specific high metabolic demands. In adults, iatrogenic hyperglycemia caused by parenteral nutrition has been studied in the context of age-related differences, but except for two case reports there are no systematic studies or case series on ISH in pediatrics [5–7].

Conditions similarly characterized by high blood glucose levels are hyperosmolar hyperglycemic syndrome and ketoacidosis, both complications of diabetes mellitus. Stress hyperglycemia in critically ill children can also go along with extremely high glucose concentrations > 300 mg/dl [3, 4, 8–10]. Once the underlying disease is treated, stress hyperglycemia tends to be resolved spontaneously [4]. No reports elucidating the pathophysiology and best management of ISH are available. The transfer of recommendations about treatment of diabetic complications to the management of ISH calls for caution as its pathophysiology may substantially differ.

A case of hyperglycemia after i.v. administration of glucose in our hospital and the lack of literature on this topic led us to initiate a survey in German, Swiss and Austrian pediatric intensive care units. Our aim was to find out more about the circumstances of iatrogenic hyperglycemia after intravenous administration of glucose-containing fluid, its treatment and outcome.

## Methods

### Patients

One hundred five pediatric intensive care units in Germany, Austria and Switzerland were identified using a database provided by the German Society of Neonatology and Pediatric Intensive Care (GNPI). The medical heads of units were contacted via email in September 2014 and asked to report any case of ISH in recent years (without specific time frame) by completing a questionnaire. Non-responding ICUs were recontacted five months after the initial email. Inclusion criterion was hyperglycemia > 300 mg/dl (16.7 mmol/l) following unintentional administration of large amounts of intravenous glucose. The study protocol was approved by the ethics committee of the Medical Faculty of the University of Duisburg-Essen (Ethik-Kommission der Medizinischen Fakultät der Universität Duisburg-Essen). Informed consent was not obtained because the study was performed retrospectively and anonymously.

### Data collection

Patient charts were reviewed retrospectively, and anonymized data transferred to the questionnaire. General data collected were the children’s hospital size, type of ward, patients’ age and gender and underlying disease.

For assessment of the circumstances of ISH occurring, day and daytime were reported, along with the cause for ISH and type and rate of infusion prescribed and administered. Data describing the course of disease include the maximum blood glucose level, hours to normal blood glucose (< 180 mg/dl [10.0 mmol/l]), hypoglycaemia (< 60 mg/dl [< 3.3 mmol/l], in neonates < 45 mg/dl [< 2.5 mmol/l]) and further laboratory parameters (serum-creatinine, −sodium, −potassium, −lactate, pH, base excess, troponin I). For laboratory data, we asked to report the most altered value within the first 24 h after detection of hyperglycemia.

To assess the severity of disease, data on mechanical ventilation, vasopressor treatment, duration of stay in the intensive care unit and survival were collected. Documented management parameters were administration of insulin, infusions, and dialysis. Complications and outcome were assessed as follows: altered mental state, minimal value on Glasgow coma scale, seizures, cerebral imaging, and persisting sequelae (conditions that first occurred during the episode of ISH but did not resolve). Finally, we asked whether and which actions for the management of i.v. solutions containing glucose had been taken after the incident.

### Statistics

Statistical analyses were performed by using Microsoft Excel® Version 16.27. Descriptive patient data were analysed for the whole cohort (median and range) and a selection of parameters was separately calculated for subgroups with favorable outcome (alive, no sequelae) and poor outcome (dead or persistent sequelae).

## Results

We received answers from 50 (48%) out of 105 neonatal/pediatric intensive care units, of which 42 (84%) reported that ISH had never occurred. Of the remaining eight hospitals one reported four patients, one reported three patients, and five hospitals one patient each, comprising 12 patients in total. Hospitals reporting respective cases were all from Germany. The reported cases dated back up to 13 years. One patient was excluded from analysis due to lack of inevitable data, such as blood glucose level, laboratory results, type of i.v. solution and further.

Reported incidents occurred between 2001 and 2015. Two patients (18%) were preterm infants (24 and 33 weeks corrected gestational age), one newborn (9%), four (36%) infants and five (45%) children. Five (45%) patients were male, six (55%) female. Details on individual patient characteristics are provided in Table 1. Two patients (18%) died and two (18%) experienced sequelae, forming the poor outcome group consisting of four patients (36%). For seven patients (64%) no sequelae were reported. We considered these patients to have good outcome.

**Table 1:** Patient details

	Pat. No.	Age and diagnosis	Glucose concentration and error type	Maximum serum glucose [mg/dl (mmol/l)]	Reduction of blood glucose [mg/dl/h (mmol/l/h)]	Insulin therapy	Initial pH	Most altered Na + within first 24 h of hyperglycemia [mmol/l]	Sequelae
**Good outcome**	1	Newborn, preterm birth	G12.5%, individual PN, AE: infusion rate too high (prescribed 4.5 ml/h)	1000 (55.5)	240 (13.3)	No	7.30	140	None
	2	Newborn, esophageal atresia	G40%, individual PN, AE: infusion rate too high (prescribed 1 ml/h)	2240 (124.3)	173 (9.6)	Yes	7.07	127	None
	3	Infant, craniosynosthosis	G50%, AE: 33 ml/h instead of 3.3 ml/h	595 (33.0)	210 (11.7)	Yes	7.38	134	None
	4	Child, after liver transplant	G20%, AE: bolus of glucose instead of cristalline solution	539 (29.9)	75 (4.2)	No	7.36	149	None
	5	Child, after surgery	G50%, AE: infusion rate too high	800 (44.4)	–	–	–	–	None
	6	Child, rhabdomyo-sarcoma	G70%, individual PN, error type unknown (prescribed infusion rate 24 ml/h)	983 (54.6)	91 (5.1)	Yes	n.d.	–	None
	7	Child, acute lymphatic leukemia	ME: wrong composition of individual PN	840 (46.6)	113 (6.3)	Yes	6.96	163	None
**Poor outcome**	8	Newborn, preterm birth	G50%, individual PN, AE: infusion rate too high (prescribed 2.4 ml/h)	569 (31.6)	58 (3.2)	Yes	7.03	142	CP, psychomotor retardation
	9	Infant, biliary atresia	Unknown glucose concentration, AE: individual PN, infusion rate too high (prescribed 15.8 ml/h)	1810 (100.5)	72 (4.0)	No	6.74	118	Died
	10	Infant, gastroenteritis	G50%, PE: prescribed infusion rate too high	> 2000 (> 111.0)	–	Yes	n.d.	–	Died
	11	Child, after bone marrow transplant	70% glucose, AE: 350 ml in 2 h instead of 30 ml/h	1900 (105.5)	205 (11.4)	Yes	7.02	159	Chronic renal failure

*G* Glucose, *PN* Parenteral nutrition, *CP* Cerebral palsy, *AE* Administration error, *PE* Prescription error, *ME* Mixing error

Patients in the poor outcome group were younger compared to the good outcome group (Table 2). A comparison of the two outcome groups (poor vs. good) is provided in Table 2).

**Table 2:** Comparison of clinical parameters by outcome

	All patients	Poor outcome	Good outcome
Number of patients n (%)	*n* = 11 (100%)	*n* = 4 (36%)	*n* = 7 (64%)
Age group	3 newborns	1 newborn	2 newborns
	3 infants	2 infants	1 infant
	5 children	1 child	4 children
Ward non-ICU	4/11 (36%)	3/4 (75%)	1/7 (14%)
Occurrence during night or weekend	6/10 (60%)	2/4 (50%)	4/6 (67%)
Maximum glucose > 1500 mg/dl (83.3 mmol/l)	4/11 (36%)	3/4 (75%)	1/7 (14%)
Reduction of blood glucose > 100 mg/dl/hour (> 5.6 mmol/l/h)	5/9 (56%)	1/3 (33%)	4/6 (67%)
Hours to normoglycemia [median (range)]	7 (2–23) (n = 9)	8.5 (7–23) (*n* = 3)	6 (2–12) (n = 6)
Dysnatremia (Na^+^ < 130 or > 145 mmol/l)	5/8 (63%)	2/3 (67%)	3/5 (60%)
Dyskalemia (K+ < 3.5 or > 5.0 mmol/l)	6/9 (67%)	3/3 (100%)	3/6 (50%)
pH < 7.1	5/8 (63%)	3/3 (100%)	2/5 (40%)
Base excess < −10.0 mmol/l	3/8 (38%)	2/3 (67%)	1/5 (20%)
Lactate > 4 mmol/l	5/6 (83%)	2/2 (100%)	3/4 (75%)
Mechanical ventilation	8/11 (73%)	4/4 (100%)	4/7 (57%)
Vasopressor therapy	5/9 (56%)	2/3 (67%)	3/6 (50%)
Insulin therapy	7/10 (70%)	3/4 (75%)	4/6 (67%)
Dialysis	2/11 (18%)	2/4 (50%)	0/7 (0%)
Coma	5/8 (63%)	3/3 (100%)	2/5 (40%)
Seizures	5/11 (45%)	3/4 (75%)	2/7 (29%)

### Circumstances of ISH

Eight (73%) children were treated in an intensive care unit (ICU), three (27%) patients on a peripheral ward. ISH was detected three times (27%) during late shift on a weekday, three times (27%) during daytime and four times (36%) during night shifts at weekends. Time of occurrence was not reported in one case. In ten (91%) cases, the i.v. solution contained glucose concentrations of 20% or more. In one case (9%) the i.v. solution contained 12.5% glucose. Administration errors were the reason in eight cases (73%), in one (9%) case the i.v. solution was incorrectly mixed, in one (9%) case wrongly prescribed, and in one case the reason remained unreported.

### Course of disease and laboratory results

Maximum blood glucose level was at median 983 mg/dl (54.6 mmol/l) (range 539–2240 mg/dl; 29.9–124.3 mmol/l). Normoglycemia (< 180 mg/dl [10.0 mmol/l]) was achieved after 7 h (range 2–23, *n* = 9). The drop of blood glucose was 113 mg/dl/h (6.3 mmol/l/h) at median (range 58–240 mg/dl/h; 3.2–13.3 mmol/l/h). Hypoglycemia (24 mg/dl [1.3 mmol/l]) was reported in one out of 9 patients. This patient did not receive insulin. Sodium levels were 141 mmol/l at median (range 118–163 mmol/l, *n* = 8). Reported Creatinine levels had a median of 0.84 mg/dl (range 0.22–1.90 mg/dl, *n* = 6). Potassium was 4.6 mmol/l and ranged between 2.5 and 6.3 mmol/l (n = 9). Lowest pH was at a median of 7.05 and ranged between 6.74 and 7.38 (n = 8), while base excess was reported with a median of − 9.3 mmol/l (range − 20.4 to − 4.0 mmol/l, n = 8). Lactate was 13.3 mmol/l at median (range 2.8–22.3 mmol/l, n = 6).

### Severity of disease

Six patients (55%) required intubation and mechanical ventilation after detection of ISH, two further patients (18%) were already ventilated at its detection. In total, eight patients (73%) obtained mechanical ventilation during ISH. Vasopressor therapy was initiated in five patients after detection of hyperglycemia (50%, *n* = 10). Median ICU stay was 6 days (range 2–90 days, *n* = 9). Two patients (18%) died.

### Therapeutic measures

Insulin therapy was started in seven of ten patients (70%). The type of i.v. solution administered following detection of ISH was recorded in eight patients, five (63%) of whom received glucose-containing solutions and three (38%) glucose-free solutions, respectively. Dialysis was initiated in two patients (18%), of whom one (9%) did not show recovery of renal function.

### Complications and outcome

Coma was reported in five (56%) of nine patients, one patient (11%) was sedated when ISH was detected. Glasgow coma scale values were only reported for two patients, inhibiting further analysis. Five patients (45%) showed seizures. Cranial imaging was performed in seven (70%) of ten reported patients. Three patients without respiratory or neurologic symptoms did not receive any imaging. In two (20%) of the reported ten patients no abnormality was detected by cerebral ultrasound. Cerebral computed tomography was performed in five patients (50%). Two patients (20%) showed generalized cerebral edema, whereas intraventricular hemorrhage, frontal hygroma and intracerebral hemorrhage was found in one patient each. Of the deceased children, one showed intracerebral hemorrhage and one generalized cerebral edema. Cause of death was not reported.

Sequelae were reported in two patients (18%). One patient developed cerebral palsy and developmental delay. One patient developed end-stage renal disease requiring ongoing dialysis.

### Actions taken

In the survey, the question about steps implemented as a consequence of the incident was answered in six cases (55%). In two cases, no actions were taken as a result of the incident. In one case, a protocol was written, and the parents were informed. In another case the programming of the syringe pumps was changed, and in one case a report was made to the critical incident reporting system and doublecheck was established. In the case of incorrectly mixed nutrition, the hospital’s pharmacy implemented a two-man-rule for manufacturing of parenteral nutrition.

## Discussion

Iatrogenic severe hyperglycemia is a rare and potentially fatal event with little knowledge about treatment and outcome. Herewith, we report the first case series in pediatric patients from an international retrospective survey, showing that ISH can lead to severe disease. Our cohort suffered high mortality and high morbidity including long-term sequelae, likely resulting from the underlying diseases combined with complications induced by ISH. Moreover, a striking number of patients required invasive treatment including mechanical ventilation and vasopressor support. The majority of patients presented metabolic acidosis and electrolyte imbalances along with neurologic symptoms such as coma and seizures.

A study investigating pediatric emergency patients with stress hyperglycemia > 300 mg/dl (16.7 mmol/l) reports a significant correlation between blood glucose levels and mortality [4]. Severity of disease, metabolic derangement, and mortality were similar to our case series [4]. Stress hyperglycemia was partially iatrogenic in about 65% of cases and was quickly resolved by treatment of the primary illness without need for insulin administration [4]. Insulin production and sensitivity are preserved in patients with iatrogenic hyperglycemia. Thus it may be possible to abstain from insulin therapy once the administration of glucose is discontinued and wait for glucose levels to drop spontaneously. Nevertheless, close monitoring of blood glucose levels appears necessary. In our small case series, time to normoglycemia was longer in patients with poor outcome than in patients with favorable outcome. A reduction of serum glucose of more than 100 mg/dl/h [5.6 mmol/l/h] is considered a risk factor for brain edema due to fluid shifts between intra- and extracellular matrix in diabetic ketoacidosis [1, 2, 11]. As this occurred in both outcome groups, rapid normalization of serum glucose alone does not explain the differences in outcome but is possibly influenced by the duration of hyperglycemia and accompanying metabolic and electrolyte imbalances, as well.

Contrary to diabetic ketoacidosis and hyperosmolar hyperglycemic state, patients are not generally dehydrated when ISH occurs. As the most common reason for ISH in the cohort was a falsely installed and thus too high infusion rate, patients are likely to suffer fluid overload and may experience additional metabolic or electrolyte imbalances depending on the type of infusion. This is concordant with our finding that numerous patients suffered from metabolic acidosis and electrolyte derangement.

Neurological complications occurred frequently: Patients with poor outcome presented coma and seizures more often compared to those with good outcome. The present data do not allow to distinguish whether the complications were the cause or the result of severity of disease, but the frequent finding of neurologic symptoms and complications points out the importance of close neurological monitoring in patients with ISH.

However, prevention is even more important than management and monitoring of ISH. In this cohort, most incidents occurred in intensive care units with i.v. solutions containing high glucose concentrations. On the other hand, all patients who experienced ISH on a peripheral ward suffered poor outcome and had very high blood glucose levels. A better surveillance in ICUs may lead to earlier detection and treatment of hyperglycemia. ISH occurred during late or night shift or at weekends in all cases. This finding might be related to a lower staff/patient ratio that is typical in German hospitals during these shifts. Constant staff/patient ratios could help to prevent miscalculation and false programming of administration rates. An effective strategy to prevent incorrect mixing of glucose solution is the two-man rule, meaning a compulsory double-check routine of important medical procedures. As a matter of fact, this rule was implemented in the corresponding hospital as a consequence of the incident reported here. The two-man rule can also be applied to confirm correct administration rates of i.v. solutions. Other prevention strategies highlighted in current literature include the implementation of CIRS (critical incident reporting system) and the use of computerized physician order systems and electronic health records [12–15]. In several cases, the prescribed infusion rates contained decimal places, which can easily lead to the administration of tenfold rates when infusion pumps are programmed erroneously. To prevent this, our children’s hospital, no longer allows programming of infusion pumps for parenteral nutrition and standard i.v. solutions with decimal places (except for the neonatal wards). Whenever possible, the administration of readily prepared solutions can help to reduce the risk of wrong mixing. Also, avoiding the use of high glucose concentrations can prevent or at least attenuate the effects of administration errors. In children that require volume restriction, the lowest possible glucose concentration should be selected. In any case, special attention of both doctors and nurses is necessary when administering i.v. solutions that contain more glucose than the age specific standard solutions.

The low number of cases in relation to the total number of cases treated in 50 hospitals during the 13-year study period and the heterogenous cohort regarding age and basic diseases contribute to the major limitations of this study. Treatment errors are still a sensitive subject in health care and probably not all cases were reported. We cannot completely rule out confounding based on comedication, the route of sampling or interfering substances, as has previously been reported [16]. Additionally, frequency of monitoring glucose levels was not standardized between institutions. However, the high rates of invasive therapies and adverse outcome highlight the need for valid data about incidence, circumstances, best management, and complications of ISH. A prospective multi-centered approach is inevitable in order to learn more about this condition.

## Conclusion

In conclusion, this is the first case series about ISH caused by parenteral administration of glucose in pediatric patients. Overall risk factors for morbidity and mortality cannot be derived from this study, but the results show that ISH is a severe condition with high morbidity and mortality that frequently requires invasive therapeutic measures. Prospective studies are needed to develop treatment strategies, along with measures for prevention.

## Data Availability

The generated data sets are available from the corresponding author on reasonable request.

## Abbreviations

CIRS: Critical incident reporting system
ICU: Intensive care unit
ISH: Iatrogenic severe hyperglycemia
i.v.: Intravenous
